# Draft Genome Sequences of Three *Bacillus* Species from South African Marine Sponges

**DOI:** 10.1128/genomeA.00143-16

**Published:** 2016-04-07

**Authors:** Leonardo Joaquim van Zyl, Relebohile Matobole, Fleury Augustin Nsole Biteghe, Timothy Klein, Bronwyn Kirby, Marla Trindade

**Affiliations:** Institute for Microbial Biotechnology and Metagenomics (IMBM), University of the Western Cape, Bellville, Cape Town, South Africa

## Abstract

The rise in antibiotic-resistant bacteria has spurred efforts to identify novel compounds with antimicrobial activity. This brief report describes the genome sequence of three *Bacillus* species isolates from South African marine sponges, which produce compounds with antimicrobial activity. A search for secondary metabolite clusters revealed several secondary metabolite pathways in these genomes, which may hold promise as novel antibiotics.

## GENOME ANNOUNCEMENT

Here, we present the draft genome sequences of three *Bacillus* spp. (PE5-112, PE8-15, and PE8-121b), which were isolated from marine sponges. The two sponges, *Spongia* sp. strain 001RSASPN and *Waltherarndtia caliculatum*, were collected in Algoa Bay off the coast of Port Elizabeth in the Eastern Cape region of South Africa. The *Bacillus* species were isolated as part of a screening program for bacteria that produce compounds with novel bioactivities. PE8-15 shows antibacterial activity toward *Escherichia coli* 1699 (resistant to 51 known antibiotics; obtained from Cubist Pharmaceuticals), *Pseudomonas putida* ATCC 27853, *Staphylococcus epidermidis* ATCC 14990, *Bacillus cereus* ATCC 10702, and *Aspergillus fumigatus* ATCC 46645. PE8-121b has activity against *B. cereus* and *Mycobacterium tuberculosis* H37RA, while PE5-112 has activity toward *Mycobacterium smegmatis* LR222, *B. cereus*, and also displays anticonvulsant (photomotor response) properties in a zebrafish model.

Genomic DNA was prepared using standard proteinase K-SDS treatment and then purified using phenol-chloroform extraction. The DNA was further purified using the Qiagen QIAex II gel extraction kit prior to sequencing. Sequencing was performed on an Illumina MiSeq, using the Nextera XT kit for library construction and MiSeq reagent kit V3 (2 × 300 bp). Genome assembly was performed using CLC Genomics Workbench version 6.5. The raw reads were trimmed and demultiplexed, and contigs ≤500 bp were removed from the final assembly. The genome sizes are ∼5.91 Mbp (35.1% G+C content), ∼5.65 Mbp (35.2% G+C content), and ∼5.36 Mbp (37.9% G+C content) for PE8-15, PE8-121b, and PE5-112, respectively, with average coverages of 118-, 28-, and 311-fold. The *N*_50_ values were 97,016 bp, 110,772 bp, and 486,773 bp, respectively. The closest relatives based on 16S rRNA gene analysis were *Bacillus mycoides* (PE8-15), *B. cereus* (PE8-121b), and *Bacillus megaterium* (PE5-112). A basic annotation was performed using the RAST server (1). The numbers of predicted coding sequences are 6,092 (1,945 hypothetical), 5,874 (1,903 hypothetical), and 5,552 (1,863 hypothetical), respectively. antiSMASH 3.0 predicted a total of 5 bacteriocin, 3 microcin, 3 siderophore, 4 nonribosomal peptide synthase, 1 type III polyketide synthase (PKS), 5 terpene, 1 lassopeptide, 1 sactipeptide, and 1 hybrid (NRPS-ladderane) pathway(s) from the three genomes combined (2). The 3 siderophore pathways appear to be conserved in two of the isolates (PE8-15 and PE8-121b), with the same pathway in PE5-112 showing areas of similarity interspersed with regions of novel sequence. While a bacillibactin-related NRPS is shared between PE8-15 and PE8-121b, it is not present in PE5-112. The type III PKS is unique to PE5-112, while the NRPS-ladderane hybrid and lassopeptide are unique to PE8-15. These genomes might be of interest to those looking to identify the pathways used to produce novel bioactive compounds.

### Nucleotide sequence accession numbers.

These whole-genome shotgun projects have been deposited at GenBank under the accession numbers LRPG00000000, LRPH00000000, and LRPI00000000 for PE5-112, PE8-15, and PE8-121b, respectively.
